# Supplementary material for: Are carbon emissions trading and green financial instruments synergistic? -Comprehensive quantitative research based on content analysis
Source: PLoS One. 2024 Mar 7;19(3):e0298601. doi: 10.1371/journal.pone.0298601 (PMC10919601; doi:10.1371/journal.pone.0298601)
Supplement: S1 Data — (ZIP) [file pone.0298601.s001.zip › Data/地级市Z_F工作报告.zip!/╔╜╢1⁄2/╝├─■╩╨/└┤╫╘╣1⁄2╓┌║┼í╛┬φ┐╦╩2╛▌═°í┐_2014.docx]

现在，我代表济宁市人民政府向大会报告工作，请予审议，并请市政协委员和其他列席会议的同志提出意见。

　　一、2013年工作回顾

　　过去的一年，面对复杂多变的外部环境和经济下行的内在压力，在中共济宁市委的正确领导下，全市上下按照“落实年、突破年”总要求，综合实施系列创新性政策措施，全市经济发展稳中有进，社会事业全面进步，百姓福祉持续提升。预计全市地区生产总值完成3501亿元、增长11%；公共财政预算收入达到302.2亿元、增长13.3%；全社会固定资产投资2110亿元、增长23%；社会消费品零售总额1470亿元、增长13.2%；城市居民人均可支配收入27948元、农民人均纯收入11300元，分别增长9.8%和13%，较好地完成了全年任务目标。（一）坚持抓转型稳增长，工业经济持续向好。纵深推进工业强市战略，实施“1351”百千亿产业培植工程，“一企一策”解决重点企业困难问题，工业经济实现增量提质。规模以上工业企业新增475家、达到1950家；工业增加值增长12.5%，制造业占工业的比重达到57%，战略性新兴产业主营业务收入增长34%，高新技术产业占比提高到24%，我市成为首批“国家信息消费试点城市”，济宁高新区高效传动与智能铲运机械产业集群被确定为国家创新型产业集群试点。全市销售收入过百亿企业达到9家，4家企业上榜中国企业500强。在全省首创小额贷款保证保险，成功发行首期“区域集优”中小企业集合票据，辰欣、润峰等骨干民营企业加速成长，泰丰液压、卡松科技等科技型中小企业活力迸发。全民创业行动深入开展，民营经济实现增加值2540亿元、增长16%。（二）坚持抓基础促增收，农业综合生产能力显著增强。粮食生产实现“十连增”、总产达到115.6亿斤。新增市级农业产业化龙头企业93家、“三品一标”产品129个，标准化生产基地发展到283万亩。新增家庭农场574家、农民合作社3782家，土地流转154万亩，农业机械化水平达到86%，大田作物土地托管服务模式在全国推广，济宁农高区晋升为国家农业科技园区。渔业转型升级六大工程和畜牧业生态健康养殖成效明显。现代水利示范市建设加快推进，全国小型农田水利重点县实现全覆盖，解决了26.2万人饮水安全问题。兑现各项惠农补贴9.5亿元，新一轮扶贫开发实现14万人脱贫。（三）坚持抓调整优结构，服务业实现增量提质。完成投资930亿元、占比达到44%，增加值占比提高1个百分点，研发设计、金融保险、软件和信息服务等生产性服务业加快发展。国家创新型试点城市建设扎实推进，取得重要科技成果162项、专利授权5500件，新引进创新创业团队10个、国家千人计划专家5名，新建院士工作站8家，新增省级以上工程技术研究中心8家、企业技术中心12家、国家级高新技术企业56家，工程机械、生物医药等十大公共技术平台功能不断提升。全市创业基金发展到14支、规模40亿元。通过发行企业债券、中期票据实现直接融资120亿元。新开业银行机构32家，其中村镇银行3家。金融机构年末存贷款余额分别达到3561.1亿元、2276.5亿元。曲阜文化经济特区规划建设扎实推进，尼山圣境、东方文博城等98个文化旅游大项目加快建设，全市实现旅游社会总收入389亿元、增长14%。济宁曲阜机场开通新航线8条，港航吞吐量4883万吨，公路货运量2.46亿吨，现代物流对经济发展的拉动作用明显增强。（四）坚持抓项目蓄后劲，县域实现争先进位。持续开展境内外系列招商活动，加强与世界500强、大型央企和知名民企对接合作，到位市外国内资金790亿元、增长30.2%，实际利用外资8.1亿美元、增长5.2%，新引进美国惠普等3家世界500强企业，26个央企合作项目开工建设。新上亿元以上项目376个，玉柴发动机、小松大型矿用车等一批产业项目竣工投产。深化与省直部门战略合作，争取上级各类转移支付资金121.4亿元。园区服务功能明显增强，14个省级经济园区销售收入、公共财政预算收入增幅均高于全省平均水平。县域整体实力大幅提升，市直部门落实帮扶项目250个、资金15亿元，市财政一般性转移支付116.7亿元；县域公共财政预算收入、固定资产投资增幅分别高于全省平均4.7、3.2个百分点；6个县市区在全省县域公共财政预算收入排名中位次前移，最高的前移10个位次。（五）坚持抓融合强功能，城乡面貌变化显著。深入实施城镇化追赶战略，城镇化率提高2.2个百分点，达到48.3%。新开工城建项目247个，完成投资369.5亿元，27个省运会系列场馆建设主体竣工。“三河六岸”开发提速推进，11公里滨河大道升级改造任务全面完成，“运河之星”等旗舰项目启动建设。新增供热面积158万平方米、天然气用户1.15万户，城区垃圾无害化处理率达到100%。都市区城际公交、济宁城区至高铁曲阜东站快速公交开通运营。滨湖大道济宁至鱼台段、济宁北二环、东外环南延和高铁曲阜东站连接线建成通车，济徐高速济宁至鱼台段、滨河大道济宁至梁山段开工建设，都市区交通一体化进程加快。县城和小城镇集聚功能、承载能力大幅提升。（六）坚持抓整治保生态，环境质量明显改善。持续抓好水污染防治，119个国家治污项目提前建成运行，13个入湖考核断面及5处南四湖监测点位水质全部达标，为实现南水北调东线一期工程顺利通水作出积极贡献。重拳整治大气污染，SO2浓度均值、PM10同比改善22.7%、3.9%，大气环境质量改善幅度居全省前列。实施108项节能技术改造、100个重点节能项目，淘汰70家企业落后产能，超额完成节能减排任务。启动十大绿化工程，新增造林24.9万亩，完成湿地保护修复20万亩，治理采煤塌陷地2.1万亩，整治土地30万亩，新建国家级森林公园、湿地公园2处，微山湖湿地入选“中国十大魅力湿地”，泗水成功创建全国绿化模范县，建成省级生态示范县6个、示范乡镇28个。（七）坚持抓民生增和谐，群众得到更多实惠。扎实推进民生保障十大体系建设，全市公共财政民生支出238亿元，占财政支出的56%。为民所办10件实事如期兑现，群众满意度持续提升。就业方面，市进城务工人员服务中心投入使用，一刻钟就业服务圈基本形成，就业困难群体实现托底安置，新增城镇就业8万人、农村劳动力转移就业8.5万人。社保方面，社会保险费实现一票征缴，连续九年提高企业退休人员养老保险金待遇。城镇居民大病保险、城镇职工居民医疗费最高报销额度均提高到25万元，新农合医疗费最高补偿额度达到15万元，生育保险政策范围内医疗费用实现个人零负担。城乡低保持续扩面提标，农村低保对象新增4.1万人。创新特困家庭救助和社会化养老服务机制，救助城乡困难群众3.8万人，新增养老服务床位1.1万张。教育方面，济宁一中太白湖校区投入使用，新改建公办幼儿园296个，竣工校安工程1374个，新增市级规范化学校29个；发放学生营养补助金1.17亿元，受惠学生66万人次。卫生方面，“先看病后付费”诊疗服务模式在全市所有定点医疗机构推开，累计受益群众262万人次，免费开展孕妇产前筛查和农村适龄妇女“两癌”筛查，惠及102万人。残疾人康复服务和保障水平明显提高。住房方面，开工建设保障性住房14689套，改造城市棚户区4318套、农村危房1.2万户。文化方面，文明城市创建扎实推进，“十艺节”两大主体赛事综合成绩全省第三，《圣水河的月亮》荣获“文华奖”，百姓大舞台建设工程被评为第二批国家公共文化服务示范项目，曲阜、邹城入选首批“省级公共文化服务体系示范区”，大运河南旺枢纽工程顺利通过世界申遗专家评估。省运会、省残运会赛事筹办和备战参赛进展顺利。完成市县食品药品工商质监管理体制改革任务。安全生产形势持续稳定，基层治安防控体系日益健全，“干部联系农户”活动扎实开展，信访责任制和领导干部公开接访制度深入落实，各类矛盾纠纷调处及时有效，社会应急管理切实加强，我市被评为“全国社会管理综合治理优秀市”。双拥创城顺利通过省考评验收，国防动员、民兵预备役工作富有成效。第七批援疆任务圆满完成。国家安全、新闻出版、广播影视、民族宗教、公共资源交易、住房公积金、外事侨务、统计、物价、粮食工作取得新成绩。档案史志、人防地震、水文气象、人口计生、妇女儿童、慈善、红十字等项事业加快发展。

　　一年来，我们以建设人民满意的服务型政府为目标，不断加强政府自身建设，主动接受人大依法监督、政协民主监督，人大代表建议、政协委员提案办复率均为100%。制定重大事项决策暂行办法实施细则，政府投资项目审计监督和公共资源交易监管力度加大。首次公开市级财政和54个市直部门“三公经费”预算。严格落实中央八项规定，大力改进工作作风，政风行风大为改观。

　　各位代表，过去的一年极不平凡。作为典型的煤炭资源型城市，面对煤炭量价齐跌、效益大幅下滑的巨大压力，各级各部门精心运筹、沉着应对，经济发展实现了稳中向好，许多工作呈现出新亮点、新突破。一是招商引资实现新突破。投资20亿美元的惠普项目落户我市，这是改革开放以来济宁引进的最大外资项目，对我市转型发展将产生重大而深远的影响。二是行政区划调整实现新突破。设立新任城区、兖州区、济宁经济技术开发区和商贸物流园区，济宁高新区、太白湖新区实现扩容升级，市区行政区域面积由884平方公里增加到1534平方公里，人口由113.8万增加到177.5万。三是城乡环境整治实现新突破。以“五城同创”为抓手，治理城区背街小巷78条，改造旱厕213个，顺利通过省级卫生城市复审；　完成1928个村庄、6875公里道路硬化工程，新建垃圾中转站62座，城乡环境面貌焕然一新。四是营商环境实现新突破。全年共压减市级行政审批事项97项，免征市级行政事业性收费105项，成为全省审批事项、收费项目最少的市之一，各窗口单位办事效率、服务质量明显提高，赢得了广大群众和投资者的充分肯定。

　　各位代表，过去一年取得的成绩，是省委、省政府和市委正确领导的结果，是市人大、市政协和社会各界监督支持的结果，是全市上下攻坚克难、拼搏实干的结果。在此，我代表济宁市人民政府，向全市工人、农民、知识分子和干部，向各级人大代表、政协委员，各民主党派、工商联、各人民团体和各界人士，向中国人民解放军驻济部队指战员、武警官兵和公安干警，向中央、省驻济单位，向支持参与济宁建设发展的港澳台同胞、海外侨胞和国际友人，表示衷心感谢和崇高敬意！

　　我们也清醒地认识到，当前我市经济发展不可持续的问题还比较突出，尤其是在经济下行压力加大的形势下，部分企业经营困难、效益下降，稳增长任务艰巨；人均经济规模和财力偏低；经济结构性矛盾还没有根本解决，高新技术产业和服务业占比低于全省平均水平；经济发展和资源环境约束的矛盾凸显，人民群众对大气质量还不满意；城镇化水平不高，县域发展不平衡；政府职能转变还不到位，一些部门和公务人员服务意识不强，办事效率不高，作风建设需要进一步加强。对这些问题和不足，我们将采取有力措施，认真加以解决。

　　二、2014年重点工作

　　今年是深入贯彻落实党的十八届三中全会精神、全面深化改革的开局之年，也是实施“十二五”规划的攻坚之年。综观国内外经济形势，机遇与挑战并存。随着各项改革措施的落实，经济发展的内生动力和活力将充分释放；　我市各项工作部署全面展开，一批重大项目竣工投产，发展的基础更加牢固。尤其令我们深受鼓舞、倍感振奋的是，习近平总书记亲临济宁视察，对我市改革发展提出殷切期望，为我们推进科学发展跨越发展指明了方向。省委、省政府确定建设西部经济隆起带，省里出台支持济宁资源型城市可持续发展意见，曲阜文化经济特区建设进入省级战略，惠普项目落户我市，国务院、省政府批复同意我市部分行政区划调整，这些都为我们加快打造鲁西科学发展高地提供了难得机遇。只要我们坚定信心、抢抓机遇，锐意改革、攻坚克难，就一定能够在更高起点上开创崭新局面！

　　今年工作的总体思路是：全面贯彻党的十八大和十八届二中、三中全会精神，以习近平总书记视察山东、视察济宁重要讲话为指引，坚持稳中求进、改革创新、争先进位，以打造鲁西科学发展高地为统领，以“提升年、显效年”为总要求，以“五大机遇”为牵动，以工业化、城镇化“两化并进”为引擎，实施系列“三年行动计划”，更大力度推进工业、城镇、县域、文化、生态“五大突破”，加快构建民生保障“十大体系”，打好转方式调结构攻坚战，做好保障和改善民生、创新社会治理大文章，促进经济持续健康发展和社会和谐稳定，为与全省同步提前全面达小康奠定坚实基础。主要预期目标安排为：地区生产总值增长10%左右；公共财政预算收入增长12%左右；　规模以上工业增加值增长12%以上；固定资产投资增长19%；社会消费品零售总额增长13%；外贸进出口增长6%，实际利用外资稳步增长；　城镇居民人均可支配收入增长10%，农民人均纯收入增长11%、力争更高一些；城镇登记失业率控制在3%以内；居民消费价格指数涨幅控制在3.5%左右；　全面完成省下达的节能减排约束性目标；　人口自然增长率控制在9.1‰；城镇化率提高2个百分点以上。

　　实现上述任务目标，我们必须坚持深化改革，集中突破重点领域关键环节，打破体制机制藩篱，最大程度释放改革红利；必须坚持转型升级，大力实施创新驱动、人才引领战略，持续推进经济结构战略性调整，尽快实现发展方式根本性转变；必须坚持依靠群众，尊重群众的首创精神，激发群众干事创业的热情，凝聚起850万济宁人民的智慧力量；必须坚持民生优先，倾力解决群众意见诉求，让改革发展成果更多更公平惠及全市人民！重点抓好七个方面的工作。（一）着力推进产业转型，加快提升发展质量效益。一是打造工业经济升级版。把新型工业化作为实施工业强市战略的主攻方向，以工业升级版三年行动计划为抓手，全面落实十大行动任务，大力实施“1351”百千亿产业培植工程，推进工业经济扩规模增实力提质量。以惠普项目为牵动提升产业层次，加快惠普千亿级产业园区规划建设，努力在引进高端企业、高端技术、高端人才上取得实质性进展，加速形成产业集聚，打造区域信息产业高地。实施信息产业高端提升工程，推进信息技术在企业研发、生产、销售和管理等环节的应用，加快建设电子商务、工业设计等5大公共服务平台，培育6个试验区，重点支持20个两化融合项目建设。提速推进北斗产业化应用示范基地、浪潮集团云计算中心、山东物联网技术发展研究院、台联电IC设计等重点项目建设。加快改造提升传统产业，推进工程机械、化工、食品医药、纺织服装、造纸、冶金建材等优势传统产业尽快形成千亿级集群。以市场为导向，培育发展新兴产业，鼓励支持企业加强产业链高端产品研发，力争在新能源、新材料、生物医药、新一代信息技术和节能环保等重点领域实现突破，形成新的支柱产业。年内高新技术产业产值、新兴产业产值分别达到1500亿元和1000亿元，占比分别提高2个和1个百分点。加大骨干企业和中小微企业培植。确定100家重点企业纳入转型升级动态管理，实施1000项技术创新项目，完成100个、760亿元重点技改项目。完善服务企业“直通车”制度，“一企一策”为企业排忧解困。推动优势企业强强联合，跨地区、跨行业、跨所有制重组并购，扩张百亿强企方阵。实施中小企业“百家成长千家培育工程”，在企业孵化、市场准入、金融担保、科技创新、品牌建设等方面提供特别支持。深化全民创业行动，落实民营经济“六放三十条”政策规定，办好“民营经济大讲堂”，鼓励中小微企业成立产业联盟实施抱团发展。以经济园区为核心、骨干企业为依托，培育和引进上下游配套企业，拉长产业链条，推动产业集群发展。年内新增规模以上企业200家、中小微企业6000户、各类创业主体3万个以上。

　　二是大力发展现代农业。实施“粮食安全”工程。积极开展粮食高产创建，启动实施耕地质量提升计划，确保粮食总产保持在100亿斤以上。调整农业种植养殖品种和品质结构，支持发展都市农业、生态农业，建设现代渔业园区20个，生态渔业养殖面积达到35万亩，扩大畜牧业健康养殖规模，标准化养殖比重提高到62%。强化农产品质量安全监管。完善农产品质量标准、检测和认证体系，整建制推进农产品质量安全区建设，建成8个农产品质量安全监管示范县，从源头上控制农产品安全隐患。提升农业社会化服务水平。加快建立以公共服务机构为依托、农村合作经济组织为基础、农业龙头企业为骨干的新型农业社会化服务体系。积极推广大田作物土地托管服务模式，引导社会化服务组织向物资、技术、加工、销售等综合性服务领域扩展。构建新型农业经营体系。鼓励发展农村合作经济，推进家庭经营、集体经营、合作经营、企业经营共同发展，鼓励和引导工商资本到农村发展适合企业化经营的现代种养业，年内新增农业产业化龙头企业50家。强化农业基础支撑。加快农村水利工程建设，完成去冬今春1.5万项小农水和12个小型农田水利重点县建设任务。加大农业科技创新，以规划建设济宁国家级农高园为契机，集中开展良种繁育、栽培工艺等农业科技攻关，启动农业物联网技术应用示范工程，促进农业技术集成化、劳动过程机械化、生产经营信息化。积极推进扶贫开发，完成160个村、18.5万贫困农民扶贫任务。

　　三是集聚发展现代服务业。坚持把服务业发展放在优先位置，力促服务业发展提速、占比提升、结构优化、贡献提高。着力突破文化旅游业。深化旅游体制改革，搞好资源整合开发，推进旅游集团实质性运营。加快重大文化旅游项目建设，支持微山湖、水泊梁山等景区争创5A级景区。强化修学游、生态游、乡村游等精品线路整合和重点客源市场开发，以京沪高铁曲阜东站为枢纽，开通至各县市区旅游专线。大力发展现代物流业。规划建设铁路、航运、空港等物流中心，加快推进济宁西部商贸物流园区、粮食物流园区建设，培育壮大物流龙头企业，鼓励发展第三方物流，努力把济宁建成区域性物流集散地。积极发展金融保险业。加快金融集聚区建设，引进更多的金融机构落户济宁，创新发展融资租赁、资产管理等多种形式的金融业态，村镇银行实现县域全覆盖，增加社区银行网点，扩大金融业整体规模。培育发展新兴服务业。放大国家信息消费试点城市效应，加快智慧济宁时空云平台系统建设，实施物联网、云计算、大数据、三网融合等行动计划，积极发展电子商务、数字文化、连锁经营、物流配送、服务外包等新兴业态；鼓励发展中医养生、保健护理、医疗旅游等健康服务业。依据新的行政区划，优化城乡商贸网点布局，建成一批购物中心、商业街区和专业市场。强化服务业发展政策扶持，严格执行服务业优惠政策；设立服务业和旅游业发展基金，引导社会资本、生产要素投向服务业领域。年内服务业增加值占比提高1个百分点以上。（二）着力深化改革扩大开放，加快释放发展生机活力。一是全方位深化改革。认真落实中央各项改革措施，加快推进重点领域和关键环节改革。深化行政审批制度改革。正确处理政府与市场的关系，发挥市场在资源配置中的决定性作用，能由市场机制调节的一律取消审批，能交给县级审批的一律下放，能备案的一律按备案执行，努力打造行政审批“项目最少、程序最简、效率最高”的城市品牌，营造一流的营商环境。深化国有企业改革。坚持增量式改革及淘汰落后产能相结合，积极引进战略合作伙伴，实施产权制度改革，推进产权多元化，健全公司法人治理结构，激发企业内在发展活力。建立以资本管理为主的国资监管体制，增强国有资本运作和保值增值能力。鼓励民营企业参与国有企业改革，支持发展非公有资本控股的混合所有制企业，积极引导民营企业建立现代企业制度。深化财税体制改革。建立全口径政府预算体系，积极推进财政预决算、部门预算及“三公经费”公开，大幅提高一般性转移支付比例，提升基层政府保障能力。进一步扩大“营改增”试点范围。加强政府投融资管理，建立规范的债务管理和风险预警机制，严格政府举债程序，有效化解存量债务，严控政府性债务风险。深化金融体制改革。优化金融生态环境，争取建立山东（济宁）普惠金融创新发展实验区，全面启动农村信用社银行化改革，扩大投资基金、私募股权、企业债等直接融资规模，规范发展民间融资，完善多元投融资机制。建立社会信用、中小企业增信、地方金融监管、资本市场对接等服务体系。深化农村土地制度改革，全面开展农村土地承包经营权确权登记颁证，争取80%以上的村完成确权登记颁证任务，鼓励土地承包经营权在公开市场上向新型经营主体流转。完成农村集体土地所有权、宅基地使用权和建设用地使用权确权登记发证。深化事业单位分类改革。加快生产经营类事业单位转为企业或社会组织，稳步推进承担行政职能事业单位改革，全面推行公益类事业单位绩效考核。

　　二是构建开放型经济新体制。实施以质取胜和市场多元化战略，拓宽对外开放领域，建立负面清单制度，积极引进外资参与产业转型升级，进入城市建设、公用事业、商务服务和现代农业领域。主动对接自贸区建设，更多分享国家战略红利。加强与珠三角、长三角、环渤海等重点地区的经济合作，深化央企对接活动，积极引进高端大项目和资本、科技、人才等资源要素。推进市综合保税区申报工作。鼓励如意科技、山推股份等具有自主知识产权、自主品牌的企业加强出口基地建设，提高产品市场竞争力。支持太阳纸业、华勤集团等有条件的企业实施境外并购、投资合作，在参与国际竞争中发展壮大，打造源自济宁的跨国公司。

　　三是促进县域发展争先进位。深入实施“县域经济倍增计划”，所有县市区完成三年倍增目标，在全省实现争先进位。持续开展“大项目突破年”活动，年内各县市区至少新上亿元以上产业项目20个。强化园区功能和配套服务，提升各类园区的产出比重和项目集聚程度，确保增长性指标高于全市平均水平30%、约束性指标低于10%。支持兖州工业园区创建国家级开发区，其他省级园区实现晋档升级，集中培育30个特色产业园区，依靠大项目和园区经济支撑县域提速发展、跨越赶超。（三）着力拓空间提内涵，加快建设大济宁新济宁。一是加速都市区融合发展。进一步完善城市发展规划。以部分行政区划调整为契机，加快修编城市总体规划，编制完成都市区空间战略与行动计划，完善中心城区控制性详规和各专项规划，启动济宁经济技术开发区发展规划，优化组群城市空间布局，明确各板块发展定位，推动都市区组织管理、产业布局、基础设施、生态建设一体发展。进一步提升城市功能品位。实施老城区改造“三年行动计划”，改造棚户区10830户，新增供水2万户、天然气1万户。加快完善雨污分流管网、河道清淤截污等基础设施建设，改善城区河湖水系环境。合理布局学校、医院、场馆等公用服务设施，疏解老城区过密人口，增强新区发展活力。密切产城融合，放大济宁高新区辐射带动功能，建设东部大型工业集聚区，推进曲阜高铁新城建设；大力发展集航运枢纽、商贸流通、铁水联运等功能于一体的规模化临港产业，开工建设一批吸纳就业能力强、方便居民生活的城市综合体、商业中心项目，大幅提高都市区产业集聚度，增强内生动力和支撑能力，打造板块特色突出、产业错位发展、宜居宜业的现代化城市。进一步加快城建重点项目建设。突出抓好都市区道路升级改造、重点片区开发，实现路网互通互联、公共交通一体布局，年内完成滨河大道济宁至梁山段路基工程，加快济徐高速济宁至鱼台段、京杭运河北延工程建设，做好跨湖高速前期工作，开工建设西二环、北二环东延，完善都市区公交和BRT快速公交系统，构筑都市区综合交通体系。高水平推进“三河六岸”重点片区开发，加快大运河文化产业带核心区项目建设。启动泗河全流域综合开发，统一规划、分段实施，尽快把泗河建设成为连接各板块的景观带、文化带、旅游带。进一步提升城市管理水平。建立政府监督、社区管理、社会协作、市场运作、公众参与的城市管理体制，全面推行网格化管理，实现管理全覆盖、无疏漏、无死角。推行公共服务市场化改革，支持社会资本参与城市公用设施建设运营，鼓励社会力量通过委托授权、特许经营参与城市管理。

　　二是扎实推进“五城同创”。巩固提升省级卫生城市创建成果，加大治违、治脏、治乱力度，抓好环境卫生、市容市貌、市场秩序专项整治，新建农贸市场15处、公厕35所、公共自行车服务站10处，确保通过省级“除四害”达标验收。加快城市绿道、城区裸露地、生态廊道和环城森林建设，完成17条主干道绿化任务，高标准建设济宁园博园，城市绿化覆盖率达到38.5%以上。全面实施创建国家环保模范城市规划，城市环境综合整治定量考核进入全省前5名。深入开展双拥共建活动，确保通过省第二轮年度检查验收。实施市民素质培育提升工程，规范窗口行业文明行为，倡树“说文明话、办文明事、做文明人”的社会风尚，让文明新风吹遍孔孟大地。

　　三是推动县城和小城镇建设。积极探索各具特色的城镇化发展模式，有序推进县城老城改造与新城拓展，大力发展产业聚集、特色鲜明的小城镇，加快设施配套，优化服务功能，加快18个省级示范镇以及市中心镇建设，培育一批有历史记忆、有地域特色、有产业支撑的重点小城镇。落实户籍制度改革政策，尊重农民意愿，推进农村转移人口市民化。启动美丽乡村建设三年行动计划，实施“五大工程”，实现农村经济发展、乡村环境改善、农民素质提升。

　　各位代表，今年我市将承办第23届省运会和第9届省残运会，按照隆重、热烈、节俭的要求，突出“人文济宁，精彩省运”的主题，精心做好市场开发、赛会运行、环境整治、安全保障、舆论宣传等工作，以一流的场馆设施、一流的组织服务、一流的城市形象，充分展示济宁经济社会发展的辉煌成就和全市人民的良好精神风貌，努力把省运会办成一届高水平、有特色、精彩圆满的体育盛会！（四）着力推进创新驱动，加快构建区域特色创新体系。把科技创新和人才队伍建设作为城市转型的根本动力，加快国家创新型试点城市建设。一是实施企业主体创新工程。集中扶持一批科技型企业，引导企业加大研发投入、建设研发机构，重点企业研发中心覆盖面达到80%以上。支持有条件的科技型企业承担重大科研攻关任务，加大先进技术收购引进、消化吸收和再创新，提高企业集成创新和原始创新能力。深化企业与大院大所战略合作，推进产学研协同创新，年内新建校企联盟2家，新增省级以上研发机构5家，转化重大科研成果170项，实现更多企业向“创新驱动”转变。二是强化创新载体建设。深化济宁高新区与北京中关村、上海张江合作，推进华大基因济宁分院、深圳先进技术研究院济宁分院、市科技与人才创业创新孵化园建设，全年新增院士工作站3家、孵化器面积40万平方米。三是加快科技体制改革。深入落实加快科技成果转化政策，健全技术创新市场导向机制，引导创新创业基金、社会资本投向科技企业和重大项目，激发社会创新活力。建立天使基金联盟，成立济宁创投基金，搭建科技担保平台，形成完善的科技金融服务体系。加强知识产权运用和保护，促进技术成果资本化、产业化。四是大力实施人才强市战略。把人才资源作为第一资源，以高层次高技能人才队伍建设为重点，继续实施海外人才引进“511”计划和国内人才集聚“百千万”工程，加大创新创业人才和实用人才培养引进力度，加快构筑人才高地。抓好济宁高新区人才特区建设，搭建人才联盟平台，建立高端科技人才库。创新完善引才、用才、留才工作机制，培养一批富有优秀潜质的青年科技人才队伍，为打造鲁西科学发展高地提供人才支撑。（五）着力实施生态突破战略，加快建设生态文明。狠抓环境治理和生态保护。坚持“绿化当模范、治水当示范、治气当先行”，落实好生态建设三年行动计划，创造人与自然和谐共生的美丽家园。实施“蓝天”工程。严格落实分阶段逐步加严的大气污染物排放济宁标准，建立起应对雾霾长效机制。加强火电厂、水泥厂等高污染行业脱硫脱硝除尘改造。在控制能源消费总量的同时，逐步降低煤炭消费比重。建立重点区域联防联控机制，持续开展大气环境综合整治行动，加强工业废气、城市扬尘、机动车尾气、秸秆焚烧等专项治理。限行并加快淘汰黄标车。实行城区在建工程封闭式施工，严查渣土运输车辆道路抛洒行为。全面推行机械化环卫保洁，取缔违法粉煤灰厂和城区燃煤小锅炉，严格落实烟花爆竹限放措施，增加蓝天白云、繁星闪烁天数，让人民呼吸上清新的空气、享受更加舒适的环境。实施“碧水”行动。完善“治用保”防控体系，巩固扩大南水北调治污成果，分步实施“引汶补源入济”工程，积极推进农村面源污染治理，严格保护微山湖、梁济运河等重要区域生态功能，消除水质安全隐患，保证一泓清水永续北送。实施“绿地”计划。加强地质环境保护和矿区生态修复，抓好水土流失、荒山荒滩、破损山体和采煤塌陷地等生态脆弱区的恢复治理，新增造林20万亩，新建绿道300公里，完成湿地保护修复20万亩，治理采煤塌陷地2万亩，创建森林公园、湿地公园4个，建成一批省级绿化模范县、生态示范县和生态乡村。

　　加大节能减排力度。严格落实目标责任，确保完成节能减排总量控制年度目标。严把新上项目环保准入关，坚决淘汰高耗能、高污染行业和落后过剩产能。全面推行重点企业合同能源管理，实施节能降耗“双百”工程，抓好电力、建筑、交通等重点领域节能。严格执行污染物排放许可和总量控制制度，加强环境执法监管，严惩违规排污行为。大力发展循环经济，促进产业向绿色、低碳、清洁转变，建设一批绿色经济示范园区，争创国家循环经济示范市。（六）着力放大文化资源优势，加快建设文化强市首善之区。一是全面启动首善之区建设。围绕打造道德建设模范区、文明和谐示范区、儒家文化传承区，制定实施首善之区建设规划。大力推进社会主义核心价值体系建设，深入开展文明城市创建活动，全面提升市民文明素质。弘扬和传承优秀传统文化，开发一批体现儒家思想的文化产品，争取设立孔子学院总部体验基地，放大国际孔子文化节、世界儒学大会、尼山论坛对外影响，奠定济宁作为国际儒学研究交流中心的地位，努力打造儒家文化圣地和中华民族共有精神家园。二是加快曲阜文化经济特区建设。编制完成核心区发展规划，启动实施一批基础性、先导性项目。赋予曲阜文化经济特区更多自主权，鼓励在文化体制机制改革方面先行先试。加快中华文化标志城建设，实施国家大遗址曲阜片区文化遗产保护利用28个项目，抓好鲁国故城考古遗址公园、孔子博物馆等重大项目建设。三是发展壮大文化产业。深化文化管理体制改革，整合开发文化资源，推进文化资源与各类资本有效对接，与旅游产业融合发展，建立多层次的文化产品和要素市场。深化文艺院团市场化改革，加强与国内外知名文化传媒集团、演艺集团的合作，培育和引进一批传媒、演艺、出版等大型文化企业集团，创作一批文艺精品，推动优秀剧目走向市场，增强自我发展能力。发挥曲阜国家级文化产业示范园带动作用，推动嘉祥石雕文化产业园、梁山出版印刷产业园等特色园区晋档升级。加快尼山圣境、兴隆文化园、宝相寺景区、东方文博城等重点文化产业项目建设。四是构建公共文化服务体系。规划建设市文化艺术中心等重大公共文化基础设施，完成济宁杂技城主体工程。完善乡镇综合文化站、村文化大院、农家书屋和社区文化中心设施配备。常态化推进千场大戏进农村、万场演出惠民生、农村公益电影放映等文化惠民工程，公共文化设施向社会免费开放，不断丰富群众精神文化生活。（七）着力推进社会事业改革创新，持续增进百姓福祉。一是办好人民满意的教育。优化城乡中小学校幼儿园规划布局，完成城区30所中小学校幼儿园新建改建任务。实施农村义务教育校舍标准化建设工程，努力缩小校际、城乡、区域差距。进一步加强高校与地方经济社会融合发展，优化高校教育课程设置，加快现代职业教育体系建设，密切校企合作，培养更多实用型高素质技能人才。鼓励建立学校联盟，实行校长教师交流轮岗，加快教育信息化，扩大优质教育资源覆盖面。实行义务教育免试就近入学，不设重点学校重点班，努力破解择校难题。关爱学生健康成长，大幅减轻学生课业负担，加强德育体育美育教育，实现农村小学营养补助全覆盖，让每一个孩子都能快乐成长、全面发展！

　　二是实现更高质量的就业。落实政府促进就业责任，完善就业创业扶持政策，建立经济发展和扩大就业联动机制。深入实施高校毕业生就业促进计划，统筹抓好农村转移劳动力、城镇困难人员、退役军人就业。年内新增城镇就业5.7万人，农村劳动力转移就业7.2万人，高校毕业生就业率达到85%以上。创建劳动关系和谐城市，依法维护劳动者合法权益。规划建设市人力资源市场和就业创业综合服务中心，开展创业助推“1+3”活动，启动创业型乡镇、社区创建工作，努力为每一个劳动者创业就业搭建广阔舞台！

　　三是构建更加完善的保障体系。强化社会保险参保扩面、一票征缴和基金调剂，落实城乡居民基础养老金、城乡低保、农村五保和社会救助标准自然增长机制，加快社会保险由制度全覆盖向人员全覆盖转变。实施“4050”特困群体助保工程，加大特困家庭、困境儿童、残疾人救助保障力度，更加关爱农村留守儿童、妇女、老年人。加快市社会福利中心二期工程、县级社会福利中心和残疾人康复中心建设。加快构建以居家为基础、社区为依托、机构为支撑的社会养老服务体系，努力让每一个人生活得更加安心、更有保障！

　　四是提供更加优质的医疗服务。整合城乡居民基本医疗保险制度，提高政府补助标准，增加大病保险种类。深化公立医院综合改革，鼓励社会办医。加快市第一人民医院高新区分院、济宁医学院附属医院太白湖分院、市中医院新院区建设。完善“先看病后付费”诊疗模式，扩大按病种付费数量范围。实施妇幼健康提升工程，深化免费婚检、产前筛查、基因检测工作，产前筛查率达到70%。开展“万名保健医生进农户”、“中医专家服务直通车”活动，推进优质医疗资源纵向流动，更好地保障群众身心健康！

　　五是推进更加全面的住房保障。优化保障性住房类别结构，新建公租房4500套，逐步将外来务工人员纳入住房保障范围，进一步解决城镇中等偏下收入家庭、新就业人员住房困难。加快“城中村”、老旧社区改造步伐，完成省下达的农村住房建设和危房改造任务，努力实现“居者有其屋”！

　　六是创新更加有效的社会治理体制。树立系统治理理念，推动政府治理和社会自我调节、居民自治良性互动。健全基层综合服务管理平台，及时反映和回应人民群众各方面各层次利益诉求。创新社区管理，提升社区自治和管理服务水平。深化“平安济宁”建设，创新立体化社会治安防控体系，严厉打击各类违法犯罪活动。健全重大决策社会稳定风险评估机制，推进党员干部联系群众和领导干部公开接访、接听市长公开电话工作，从源头上预防和化解社会矛盾。强化安全生产红线意识，健全安全隐患排查和防控体系，严格落实政府和企业责任，坚决防止重特大事故发生。深入开展“六五”普法活动。依法管理互联网，确保信息安全。完善食品药品安全监管机构，加强监管能力、检验检测能力建设，切实保障食品药品安全。坚持计划生育基本国策，稳定低生育水平，统筹解决人口问题。加强价格调节基金征缴使用，强化粮食储备，保持物价基本稳定。加强国防动员、人民防空、民兵预备役建设。依法推进第十一届村民委员会换届选举。扎实开展第三次全国经济普查工作。统筹做好民族宗教、外事侨务、对台和老龄工作，提升住房公积金、统计、水文、防汛、气象、史志和防震减灾等项工作水平。

　　三、全面加强政府自身建设

　　打铁必须自身硬，提高政府综合素质至关重要。我们将以开展党的群众路线教育实践活动为抓手，以改革创新为统领，不断提高政府治理能力，改进工作作风，提升工作效率，努力开创政府工作新局面。

　　我们要建设法治型政府。强化法治理念、法治思维和规则意识，坚持把依法办事贯穿于政府工作的各个环节，严格按照法定权限和程序行使权力。自觉接受人大法律监督和政协民主监督，认真执行人大及其常委会各项决议决定，及时办理人大代表建议和政协提案。规范决策程序，完善议事规则，坚持重大行政决策专家论证、公众参与、风险评估、合法性审查等制度，加强重大决策跟踪反馈和责任追究。强化行政执法规范化建设，严格落实行政裁量基准制度，大力清理基层执法项目，积极预防化解行政争议，不断提高依法行政水平。加强招拍挂、公共资源交易、政府采购、财政预算制度建设，政府投资项目坚决做到“三个一律”，即未经立项的工程项目一律不准落地；　未经招投标的工程项目一律不准开工；未经论证、报批，擅自变更工程规划设计，造成工程概算超规模、超预算的一律严肃追究责任。严格落实《行政应讼办法》，健全行政复议案件审理机制，坚决纠正违法或不当行政行为，促进社会公平正义。

　　我们要建设服务型政府。强化窗口服务功能，按照应进必进、精简高效的原则，推动行政审批项目全部纳入行政服务中心办理。实施百姓办事“零障碍”工程，进一步简化事项、优化流程、按时办结，构建更加方便快捷的服务机制。加大政务公开力度，推行权力清单制度，对政府部门的职责权限、许可事项、运作流程、收费标准按规定全部公开，务求政府各项工作公开透明、阳光运行。完善政府信息发布制度，充分利用新闻发布会、政府网站、政务微博、微信等平台，及时回应群众利益关切。整合提升“12345”市长公开电话等服务热线平台，打造“线报声屏网”立体联动服务体系。切实改进会风文风，继续实行无会月制度，大幅压减会议文件，健全领导干部深入基层调查研究机制，多到矛盾困难多、群众意见大的地方去，着力解决群众所盼、企业所扰的实际问题。发扬“钉钉子”精神，雷厉风行，抓好各项工作落实。

　　我们要建设廉洁型政府。坚持用制度管权管事管人，坚决纠正“四风”，严格执行中央“八项规定”和省、市委实施办法，认真落实《党政机关厉行节约反对浪费条例》，健全行政经费支出管理制度，严禁超预算或无预算安排支出，严禁以公务差旅为名变相旅游，严禁无实质内容的学习培训和考察调研，严控公务接待、公务用车、办公用房、会议活动，年内一般性支出再压减5%。全面推进惩治和预防腐败体系建设，从源头上防治腐败。认真落实党风廉政建设责任制，加强对“三公经费”、重大项目等审计监督，支持纪检监察机关依法办案；按照“公开是原则，不公开是例外”的要求，大力度实施政务“阳光工程”；加强对政府工作人员的廉政教育和廉政文化建设，努力做到干部清正、政府清廉、政治清明。

　　各位代表，面对发展新形势、人民新期待，我们深感责任重大、使命光荣。让我们在中共济宁市委的坚强领导下，紧紧依靠全市人民，抢抓机遇、改革创新，同心同德、扎实苦干，努力打造鲁西科学发展高地，创造济宁更加美好的未来！
